# EGFR-targeted bacteriophage lambda penetrates model stromal and colorectal carcinoma tissues, is taken up into carcinoma cells, and interferes with 3-dimensional tumor formation

**DOI:** 10.3389/fimmu.2022.957233

**Published:** 2022-12-16

**Authors:** Haein Huh, Ding-Wen Chen, Marianna Foldvari, Roderick Slavcev, Jonathan Blay

**Affiliations:** ^1^ School of Pharmacy, University of Waterloo, Waterloo, ON, Canada; ^2^ Department of Pathology, Dalhousie University, Halifax, NS, Canada

**Keywords:** phage therapy, epidermal growth factor receptors, colorectal cancer, spheroids, tumor microenvironment

## Abstract

**Introduction:**

Colorectal cancer and other adult solid cancers pose a significant challenge for successful treatment because the tumor microenvironment both hinders the action of conventional therapeutics and suppresses the immune activities of infiltrating leukocytes. The immune suppression is largely the effect of enhanced local mediators such as purine nucleosides and eicosanoids. Genetic approaches have the promise of interfering with these mechanisms of local immunosuppression to allow both intrinsic and therapeutic immunological anticancer processes. Bacterial phages offer a novel means of enabling access into tissues for therapeutic genetic manipulations.

**Methods:**

We generated spheroids of fibroblastic and CRC cancer cells to model the 3-dimensional stromal and parenchymal components of colorectal tumours. We used these to examine the access and effects of both wildtype (WT) and epidermal growth factor (EGF)-presenting bacteriophage λ (WT- λ and EGF-λ) as a means of delivery of targeted genetic interventions in solid cancers. We used both confocal microscopy of spheroids exposed to AF488-tagged phages, and the recovery of viable phages as measured by plaque-forming assays to evaluate access; and measures of mitochondrial enzyme activity and cellular ATP to evaluate the outcome on the constituent cells.

**Results:**

Using flourescence-tagged derivatives of these bacteriophages (AF488-WT-λ and AF488-EGF-λ) we showed that phage entry into these tumour microenvironments was possible and that the EGF ligand enabled efficient and persistent uptake into the cancer cell mass. EGF-λ became localized in the intracellular portion of cancer cells and was subjected to subsequent cellular processing. The targeted λ phage had no independent effect upon mature tumour spheroids, but interfered with the early formation and growth of cancer tissues without the need for addition of a toxic payload, suggesting that it might have beneficial effects by itself in addition to any genetic intervention delivered to the tumour. Interference with spheroid formation persisted over the duration of culture.

**Discussion:**

We conclude that targeted phage technology is a feasible strategy to facilitate delivery into colorectal cancer tumour tissue (and by extension other solid carcinomas) and provides an appropriate delivery vehicle for a gene therapeutic that can reduce local immunosuppression and/or deliver an additional direct anticancer activity.

## Introduction

The local tumor microenvironment (TME) of a solid tumor acts to suppress anti-tumor immune responses. This is the consequence both of abnormal tissue architecture and altered cellular metabolism, particularly a tendency toward regional hypoxia as a result of the compromised vasculature (1–3).

Two of the most significant families of local mediators in this context are those deriving from purine and eicosanoid metabolism (4, 5). These two networks overlap in their impact on the factors that determine the anti-tumor immune response (6). In the case of purine metabolism, extracellular nucleotides and nucleosides released as a result of altered metabolism and enzyme levels have substantial effects on both the tumor cells and the anti-tumor immune response. The tumor microenvironment has high concentrations of adenosine (7), produced both by alteration of cellular metabolic pathways due to local hypoxia (1) and the dephosphorylation of ATP released by dying cells (8). The adenosine is produced as a result of the ectonucleotidase activities of CD73 and CD39, which may be more highly expressed in cancer (8, 9). Adenosine production is facilitated by a variety of cells within the TME, including tumor cells, regulatory T cells, Th17 cells, myeloid cells, resident mesenchymal stem cells and stromal cells (8, 10, 11).

Whereas extracellular ATP has positive effects against the tumor, adenosine has substantial pro-tumor actions (12, 13). Adenosine enhances tumor growth by acting on the cancer cells (9, 14, 15), stroma (9) and tumor vasculature (9, 16). It also favors metastasis by promoting epithelial-mesenchymal transition, migration, and invasion in cancer cells (17). We have shown that the chemokine receptor CXCR4, which regulates leukocytic and stem cell homing and cancer metastasis (18–20), and the multifunctional regulator DPP4, which controls the CXCR4 ligand CXCL12 (21, 22) are regulated by adenosine (23, 24) and selected eicosanoids (25) through pathways that we have identified (26–28).

Much of adenosine’s impact though is due to substantial local immunosuppressive actions (12). The tumor immunosuppressive effects of adenosine apply to both adaptive (29) and innate immunity (30). Adenosine-producing Treg cells are one of the several mechanisms of tumor escape from the immune system (31). Adenosine is regarded to constitute a metabolic immune checkpoint (32).

Suppression of the adenosine’s effects may be a valuable adjunct to immunotherapies in improving cancer immunotherapy (33). Targeting adenosine pathways may be useful for both solid tumours (34) and lymphocytic leukemias for which the levels of extracellular adenosine are raised (35). The actions of adenosine may be blocked either by reducing its production or by interfering with the discrete receptors through which adenosine acts on different cells, including A2a, A2b and A3 receptors (11, 36–42). The conventional approach of using systemic agents to antagonize adenosine receptors or ectonucleotidases raises a major concern, however. This is a primitive host regulatory network, and adenosine and its receptors are involved in control mechanisms throughout the body, including critical cardiovascular and central nervous system functions (16, 43). We therefore favor locally-directly ablation of excess adenosine production to remove both the stimulatory effects on the tumor population (9, 14, 15) and immunosuppression (28, 29, 39, 42). Gene therapeutic approaches are well established but have their limitations. We favor exploiting a different and novel platform, based on common inhabitants of the gut microbiome.

Bacteriophages (‘phages’) offer an alternative antibacterial strategy to conventional antimicrobial drugs and may be of enhanced value in the current antimicrobial drug-resistant era (44–48). They may be useful in combination with existing antibacterial agents (46, 49). Furthermore, their ease of manipulation and greater understanding of the properties that favor phage therapy success have raised the possibility of uses beyond infectious disease (50–52).

With the advent of phage display systems (53, 54) a wide range of macromolecular ligands may be fused to the lytic phage capsid allowing for targeted delivery of therapeutic payloads (52, 55). This technology has been applied in the design of diverse therapeutic agents (56–60) and against a range of clinical objectives (60–64). It is especially useful in the field of cancer biology, in which many delivery systems are challenged by imprecise targeting and poor penetration into the tumor interstitium (65–68).

The complex pathophysiology of the tumor microenvironment has hindered the efficient delivery of high molecular weight, targeted antineoplastic agents. Systemically-administered, targeted anti-cancer drugs need to enter the tumor vasculature network, extravasate efficiently into the interstitial space and reach a majority of the tumor cells at a pharmacologically-effective dose (66, 69–74). Achieving this has proven challenging due to the high tumor interstitial fluid pressure and an irregular vasculature that leads to tumor heterogeneity with hypoxic regions (75). Gradients of nutrients and oxygen concentrations exist in the microenvironment, resulting in more active and proliferative cells being situated closer to the vasculature. However, the tumor microenvironment becomes hypoxic (<5 mmHg pO_2_) at a distance of 70-80 µm from a blood vessel, and anoxic at 150 µm, rendering cells in these distal regions progressively more resistant to drugs that affect the cell cycle or depend on redox mechanisms (76, 77). There are also physical barriers within the tumor interstitium that may impede penetration of therapeutic drugs, such as an abnormal extracellular matrix (ECM). The fibrous protein collagen, in particular, increases in deposition with tumor progression (78, 79). Collectively, these factors constrain many anticancer agents to peripheral areas of the tumor with limited access to cells in the inner regions (67, 77, 80–83).

Phages offer advantages over both current viral and non-viral systems of targeted delivery. Their inability to multiply within human cells vastly favors phages over modified human pathogenic viruses. Moreover, in contrast to the latter oncolytic viruses, phages are unlikely to undergo alterations in their tropism, which is a major concern with the exploitation of viruses for anticancer therapy (84). In addition, phages have the ability to penetrate through epithelium and endothelium and accumulate in various mammalian tissues and organs (85, 86) as well as the ability to bypass the blood-brain barrier (85) and the gut mucosal barrier (87, 88). Vectorial transcytosis across epithelial cells has been described whereby phages can cross an epithelial cell in an apical-to-basal direction (89, 90).

The potential utility for phages is increased further as they can be engineered to enhance their application and efficacy profile (55). Tropism for specific cells can be conferred by decoration of cell-targeting ligands to phage capsid surface proteins (53). In this strategy, the gene of interest is translationally fused to a capsid gene of the phage to produce a projecting targeting ligand (91). This same phage display approach was also used early on in cancer therapy to uncover tumor-homing peptides from a library of phages (54, 92).

The cellular barriers, fluid dynamics and structure of the ECM are different between normal tissues and the tissue of a solid tumor (79, 80, 93). Therefore, there is a need to delineate the distinct capacities of phages to penetrate in an intact and functional form through both the authentic tumor interstitium and the neoplastic parenchyma. In this study we use multicellular cellular aggregates (commonly known as ‘spheroids’) to reproduce the two different local tissue environments that the phage will need to navigate in order to deliver a cancer therapeutic payload in a patient.

Colorectal adenocarcinoma (CRC) is the most common form of large bowel cancer in humans. It afflicts older patients but is showing an increasing facility to affect younger individuals, with a 7%/year increase among 15-29 year-olds in Canada (94). Our goal in this study is to understand the potential for phage to enter and deliver a genetic therapeutic to confer an antineoplastic hit in CRC. The ability to target this cancer with a strategy that does not depend on conventional mutagenic cytoxic agents also reduces the chance of secondary cancers that occur in younger patients many years post-CRC chemotherapy (95).

Firstly, we generated a spheroid model using fibroblasts, where the cellular arrangement and secreted ECM recapitulate the tight connective tissue stroma that surrounds and separates areas of neoplastic cells. Secondly, we generated human colorectal adenocarcinoma cell spheroids to model the structure of the CRC cancer parenchyma. We investigated phage access in these 3-dimensional tissue models of the CRC stroma and parenchyma with λ phage, both unmodified and with epidermal growth factor (EGF) displayed as a targeting ligand for the EGF receptor on CRC tumor cells. We first sought to understand whether unmodified phage λ can access and diffuse within multicellular spheroids generated from NIH3T3 mouse fibroblasts, representing the stroma in a solid tumor microenvironment. EGF-displaying phages were then studied to observe their interaction and targeted effect within spheroids formed from HT-29 cells, human colon adenocarcinoma cells that we have studied extensively for their abilities to respond to anticancer drugs, natural products and mediators in the tumor microenvironment (22, 23, 25, 27, 96, 97) and which are known to overexpress EGFR (98, 99).

## Methods

### Spheroid culture

NIH3T3 and HT-29 cell lines were obtained from the American Type Culture Collection (ATCC, Manassas, VA. USA), have been confirmed for genotype, and cultures were regularly tested for mycoplasma using a PCR-based approach and found to be negative for contamination. Cells were grown routinely in T25 cell culture flasks (Nunclon Delta™, ThermoFisher, Mississauga, ON) with Dulbecco’s modified Eagle medium (DMEM, HyClone™, ThermoFisher) containing 4mM L-glutamine and 4500mg/L glucose, 1mM sodium pyruvate, and 5% v/v fetal bovine serum (FBS) and incubated in a humidified atmosphere at 37°C and 90% air/10% CO_2_. Culture medium was replaced every 3 d. At 90% confluency, NIH3T3 or HT-29 monolayers were trypsinized (0.25% trypsin-EDTA) into single cell suspensions, rinsed and cells were counted with a hematocytometer (Fisher Scientific).

Spheroids were grown as previously described (97). Unless otherwise stated, NIH3T3 fibroblast spheroids were generated from 3 x 10^4^ cells for initial seeding and were cultured for a total of 10 d with medium supplementation as needed. HT-29 colon adenocarcinoma spheroids were generated from 5 x 10^3^ cells and were cultured for 7 d. Spheroids were observed with a bright field inverted microscope (Nikon^®^ TE200), and images were captured using a Micropublisher 5.0 RTV camera with QCapture Pro™ 7 software.

### Generation of EGF-displaying λ bacteriophages

Phage λ possesses approximately 415 copies of the major coat protein gpE and between 405-420 copies of gpD on the capsid surface, of which gpD is robustly tolerant of peptide fusions (53, 100). Amber suppressor strains (SupD, E, F) of W3101 *E. coli* were constructed as described (101) (**Supplementary Table S1**). Briefly, these isogenic suppressor strains of *E. coli* were transformed with a multicopy plasmid pHH1 (pPL451-gpD::EGF) to allow for the expression of *D::EGF*. The plasmid pPL451-gpD::eGFP was as described, whereby a multicopy plasmid pPL451 contained the *D::eGFP* sequence linked by an in-frame short linker of amino acids TSGSGSGSGSGT and a KpnI cut site (101, 102). In order to construct the multicopy plasmid pHH1, the sequence encoding *eGFP* was exchanged for the gene sequence of human epidermal growth factor [BC093731.1 (103)]. This encoded a translated C-terminal *EGF* translational fusion with the gpD capsid gene.

Isogenic W3101 SupD and SupF amber suppressor *E. coli* cells were transformed by the multicopy plasmid [pPL451-gpD::EGF] by the calcium heat shock system. The *D::EGF* fusion is under the control of a temperature-sensitive λ CI857 repressor that induces gene expression at 37 °C and also confers ampicillin resistance. Transformed colonies were selected on LB ampicillin agar plates. BB4 (*supE*, *supF*) *E. coli* is a double amber suppressor in which λF7 phage does not rely on the fusion plasmid for a functional copy of gene *D.* This was used as a positive control for efficiency of plating and as a negative control for *EGF* decoration.

EGF^Hi^ phages – λF7 phage with maximally decorated surfaces – were generated at higher temperatures of 37°C using SupD *E. coli* amber suppressor host for propagation (**Supplementary Table S2**). Conversely, EGF^Lo^ were generated with SupF *E. coli* strains, resulting in reduced complementation from the plasmid expressing the *D* translational fusion with overall minimal surface decoration. Previous fluorometric analysis of gpD::eGFP decorated phages estimated approximately 147 molecules per phage for the maximal fusion (SupD, 37°C), and 89 molecules per phage generated from SupF at 37°C, from which we can infer that the decoration of EGF^Lo^ phages will be approximately 40% less than EGF^Hi^ phages (101).

Transformed SupD and SupF *E. coli* cells were grown in LB broth over night at 37°C. 10-fold dilutions of primary lysates were prepared in 1 ml of TN buffer and were added to 0.3 ml of the transformed cells (grown to OD_600_ = 0.4 at 37°C), then incubated overnight at 37°C. The next day, 10 ml of TN buffer were added on to each plate and incubated for 24 h at 4°C. The top agar was scraped and transferred to a conical tube and was centrifuged at 23,000 x *g* at 4°C for 20 min (Avanti J-E Centrifuge, Beckman Coulter, Mississauga Canada). Negative control λF7 phages generated with a double suppressor BB4 *E. coli* host that did not have surface decoration (wildtype, WT) were prepared in parallel. Phage lysates were filtered through a sterile 0.2 µm syringe filter to remove cellular debris, followed by polyethylene glycol (PEG)-precipitation (Sigma-Aldrich). Precipitated phages were resuspended in DMEM supplemented by 5% FBS and were further purified through a gel chromatography. Phage samples were standardized to a concentration of 10^9^ PFU/ml. Unless otherwise stated, 100 µl of each sample was added to each spheroid for a total of 10^8^ PFU.

Plating assays showed a decline in phage infectivity of bacterial targets with time of incubation at 37°C (**Supplementary Table S3**). However, significant (~30%) infectivity was retained for both native and EGF-displaying λF7 even at the longest time period of 48 h at 37°C. Experimental data were corrected for the change in intrinsic infectivity at 37°C as noted.

### λ phage titration and standard plaque-forming unit assays

Soft-agar overlay technique was used on fresh BB4 *E. coli* cells, as this strain generates the highest titers of λF7. A liquid culture of BB4 (300 µl) was added to 3 ml of molten top agar (Bacto Tryptone and Bacto Agar from Difco Laboratories, Sparks, MD), as well as a 100 µl volume of phage dilution in suspension.

### Fluorophore labeling of λ phage and visualization using confocal microscopy

The primary amines of wildtype λ phage or EGF-displaying phage capsids were conjugated with Alexa Fluor^®^ 488 (Thermo Fisher) according to manufacturer’s instructions. Briefly, 10^12^ PFU of PEG-precipitated phages were resuspended in 95 µl of 0.1-M NaHCO_3_ buffer (pH 8.3) and were incubated with 5 µl of dye (10 mg/ml in dimethyl sulfoxide, DMSO) at room temperature for 1 h with brief vortexing every 10 min. The volume was then brought up to 1 ml and was centrifuged for 30 min at 23,000 x *g* at 4 °C and washed once. The unbound dye in the supernatant was replaced with DMEM and 5% FBS to resuspend the Alexa Fluor^®^ 488-conjugated phages. 10^8^ PFU of phages were added to HT-29 spheroids and the spheroids incubated at 37 °C for the times shown. HT-29 spheroids were stained with 10 µM of CellTracker Red CMPTX dye™ (Molecular Probes, Invitrogen) for 1 h at 37°C prior to visualization. Spheroids were washed thoroughly with phosphate-buffered saline (PBS; 137mM NaCl, 24.8mM Tris-HCL, 5mM KCL, 0.7mM Na_2_HPO_4_, 0.5mM MgSO_4_ and 1mM CaCl_2_; pH 7.2) then transferred onto a glass bottomed 24-well plate with No.0 coverslip (MatTek, Ashland, MA). Spheroids were imaged using Zeiss LSM710 laser scanning confocal microscope (Carl Zeiss AG, Oberkochen, Baden-Wü, Germany) with 20X objective. Z-stack images were captured using Zen 2009 software (Carl Zeiss AG) from the top of the sphere to the flat base of each spheroid.

### Spheroid uptake and cellular internalization assay

Prior to the application of phages, HT-29 spheroids were treated with 5 mM of NH_4_Cl for 45 min at 23°C to prevent subsequent lysosomal degradation of internalized phages (this was not done in other assays). Spheroids were washed with PBS and treated with 10^8^ PFU of phages at 37°C. Spheroids were then washed thoroughly (a total of 7 times) with PBS to remove unbound phages, and incubated with glycine (200 mM, pH 2.0) for 10 min at 4°C to inactivate the phages *in situ*. Following two further washes with PBS at 4°C the spheroids were completely dissociated in 100 μL of 0.25% EDTA-trypsin for 20 min at 37°C and pipetted thoroughly to dissociate the cells. Suspended cells were pelleted twice by centrifugation at 7,000 x *g* for 10 min, separating out the phages recovered from the *inter*cellular spaces of the spheroids, which were recovered as the supernatant. Pelleted cells were then resuspended in 100 μL of 5 mM EDTA and subjected to 3 freeze/thaw cycles to release internalized phage particles. Residual debris was removed by further centrifugation and the supernatant set aside as the “*intra*cellular” fraction. Phages were quantified for each fraction using the plaque assay as described above, and corrected for any intrinsic loss of infectivity due to incubation at 37°C.

### Assessment of phage λ on spheroid growth

The impacts of EGF-λ and WT λ phages on the growth of HT-29 spheroids were assessed with brightfield illumination (Leica DM2000, 10X objective, Micropublisher 5.0 RTV camera) using QCapture Pro™ 7 Software. No phages were added to the control spheroids. Three separate conditions were tested with HT-29 cells and two different concentrations (10^4^ or 10^8^ PFU) of phages:

- Phages were added to HT-29 cells at the time of addition onto agarose, with a pre-incubation of 4°C for 60 min, following spheroid growth to d 7;
- Phages were added to HT-29 spheroids during the spheroid formation process after 48 h and the fate of spheroids followed out to d 20;
- Phages were added to fully-mature spheroids at d 11 and the results followed to d 16.

### MTT assay on monolayer cells

The response of HT-29 cells in monolayer culture to phages as a measure of direct cytotoxicity was assayed by the MTT assay (96). Briefly, cells were seeded in DMEM supplemented with 5% FBS in 96-well plates and incubated at 37°C, 10% CO_2_ for 72 h to allow for initial adherence. The monolayers were then treated with either 10^8^ or 10^4^ PFU of EGF-decorated or WT phages for 24 h at 37°C, 10% CO_2_. After addition of MTT the plate was incubated on a rotary shaker at 37°C for 1 h to allow for the metabolic conversion of MTT into formazan. The medium was then removed and formazan dissolved with 100 μl of DMSO per well for 30 min at room temperature. The absorbance of the plate was measured at A_492_ nm on a SpectraMax M5 Microplate Reader (Molecular Devices, San Jose, USA) with subtraction of the A_492_ of blank wells.

### ATP assay on spheroids

The effect of phages on HT-29 spheroids as a measure of presumed cytotoxicity was carried out using the CellTiter-Glo^®^ 3D (Promega) kit for 3D cell cultures according to the manufacturer’s directions. This test quantitates ATP within multicellular spheroids as an indication of the putative number of viable cells. Briefly, 4 mM of diaminocyclohexane triacetic acid was added to spheroids in multiwall plates at a 1:1 ratio with the culture medium, the contents were mixed vigorously for 5 min on an orbital shaker and then incubated at room temperature for additional 15 min. Half of the samples were transferred to a separate container and diluted by 10-fold in MilliQ H_2_O with repeated mixing to ensure homogeneity. Diluted samples were then transferred to a fresh assay well. An equal volume of CellTiter-Glo^®^ 3D Reagent was added, and the contents were mixed for an additional 2 min on an orbital shaker. Finally, the plates were incubated at room temperature for 10 min to stabilize the luminescent signal, which was then recorded on the SpectraMax M5 Microplate Reader (Molecular Devices) using the luminescence mode.

## Results

### Phage λ infiltrates fibroblast spheroids

**Figure 1:**
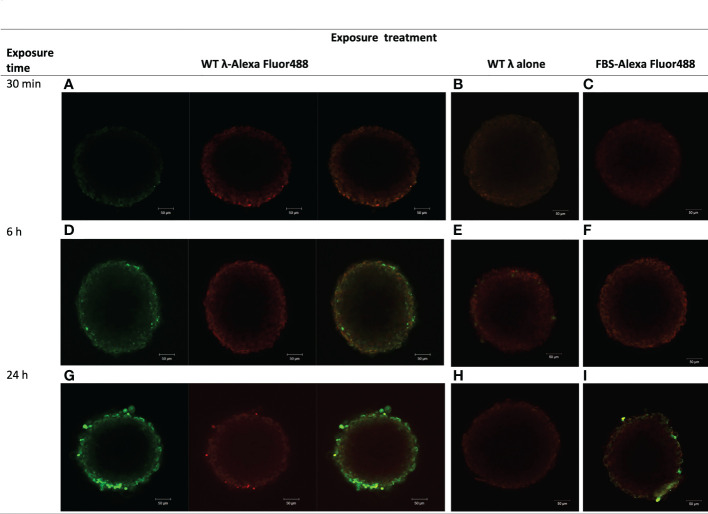
λ phages adhere to and accumulate in NIH3T3 fibroblast spheroids. NIH3T3 spheroids (d7, 250–350 μm in diameter) were exposed to either WT λ phages tagged with Alexa Fluor488 **(A, D, G)**, untagged λ phages **(B, E, H)**, or FBS tagged with Alexa Fluor488 **(C, F, I)** for the times shown. Spheroids were washed to discard unbound phages or FBS and tagging dye, and counterstained with CellTracker™ Red prior to imaging by confocal microscopy. Images are from a z plane 64 μm below the surface of the spheroids.

We first examined the ability of native (wildtype, WT) λ phages to interact with a tissue model of the tumor stroma, using Alexa AF488-tagged WT-λ phages and observing their time-dependent association with NIH3T3 fibroblast spheroids. The NIH3T3 spheroids were grown to 250-350 μm in diameter – up to the size threshold for which there may be authentic complicating effects of hypoxia in tissue due to inadequate perfusion of oxygen. AF488-WT-λ phages adhered to the fibroblast spheroids and accumulated to be resistant to washing, in a time-dependent manner (**Figure 1**). No significant fluorescence was visible after 30 min (**Figure 1A**), but by 6 h AF488-WT-λ particles were associated with the surface cells of the spheroid (**Figure 1D**), and by 24 h, the fluorescent phage showed a much more extensive association with the outer cell layers of the spheroid (**Figure 1G**). There was no significant fluorescence when NIH3T3 spheroids were exposed to either untagged WT λ phage or AF488 fluorophore alone, (**Figures 1B, C, E, F, H, I** respectively) indicating that the observed fluorescence reflected the tagged phage. Visualization of the dense core regions of the large spheroid was limited using confocal microscopy but infiltration of AF488-WT-λ phage between cells that were tightly juxtaposed within the spheroid was evident. AF488-WT-λ phage particles were evident on the peripheral cells even after spheroids had been thoroughly washed to discard weakly-bound phages, suggesting either high binding affinity or phage entrapment.

**Figure 2:**
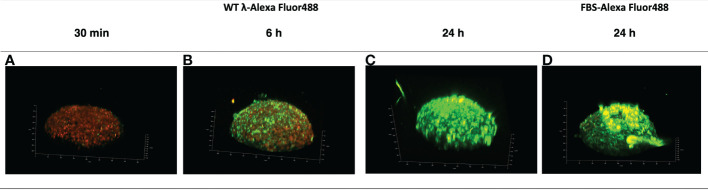
Accumulation of λ phages versus FBS at the surface of NIH3T3 fibroblast spheroids. NIH3T3 spheroids (d7, 250–350 μm in diameter) were exposed to either WT λ phages tagged with Alexa Fluor488 **(A–C)** or FBS tagged with Alexa Fluor488 **(D)** for the times shown. Spheroids were washed to discard unbound phages or FBS, and counterstained with CellTracker™ Red prior to imaging by confocal microscopy. The 3D reconstruction is shown.

**Figure 3:**
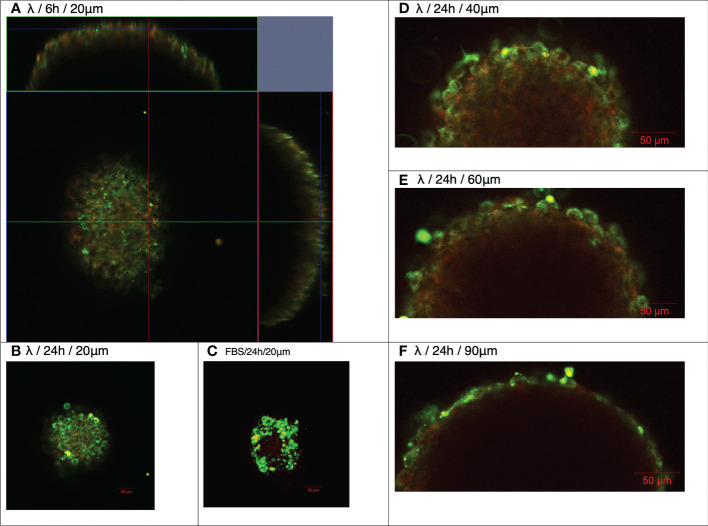
Association and penetration of λ phages with NIH3T3 fibroblast spheroids. NIH3T3 spheroids (d7, 250–350 μm in diameter) were exposed to either WT λ phages tagged with Alexa Fluor488 **(A,B, D–F)** or FBS tagged with Alexa Fluor488 **(C)** for the times shown. Spheroids were washed to discard unbound phages or FBS, and counterstained with CellTracker™ Red prior to imaging by confocal microscopy at the Z planes indicated (μm).

Examination of the localization of AF488-WT-λ phage particles at the earlier 30 min timepoint confirmed lack of association with the spheroid (**Figure 2A**). Analysis for the 6 h timepoint using either 3-dimensional reconstruction (**Figure 2B**) or orthogonal views of the spheroids (**Figure 3A**) showed that the initial association was punctate and discrete but extended over the spheroid surface. After 24 h (**Figures 2C**, **3B**) these punctate accumulations had enlarged to become similar in size to individual cells, suggesting cellular uptake of the phage. To exclude the possibility that this was not simply the result of phagocytic uptake of protein particulates by the fibroblasts, we compared with AF488-tagged FBS. Tagged FBS also associated with the spheroid surface (**Figures 2D**, **3C**) but was seen as larger conglomerates that were distributed irregularly across the spheroid surface and seemed often to be associated with dead cells or debris being extruded from the spheroid. We therefore characterize this as more nonspecific binding, in contrast to that of phage particles, which appeared more to involve specific interactions with viable fibroblasts.

Closer observation suggested that AF488-WT-λ was progressively taken up by cells at or near the spheroid surface over the 24 h incubation period. Optical slices at 20, 40, 60 and 90 μm depths (**Figures 3B, D–F**) showed individual cells that had taken up phage, in some cases retaining a cytosolic distribution, as well as vesicular structures below the surface cell layer that perhaps extended 2-3 cells deep, and a deeper, more diffuse fluorescence. Measurements of the inward radial positions of discrete fluorescent particles at deeper positions showed that the deepest vesicular structures were at: 60 μm, 20.7 ± 4.2 μm; 90 μm, 13.9 ± 4.0 μm (mean ± SD, n=4). This suggests that WT-λ packaged in cellular structures is mostly in the more surface cells after 24 h, predominantly within the first 1-3 superficial cellular layers, but that released phage or processed fragments may diffuse further. The degree of inward access over longer time periods needs further study.

### Phage λ tolerates gpD::EGF fusions in capsid assembly

λF7 phage harbors an amber *Dam15* mutation in the gene encoding the gpD capsid protein; generation of λF7 in poor or Sup^—^ *E. coli* amber suppressor hosts would lead to a greater relative complementation by the *D::EGF* fusion, resulting in increased incorporation of gpD::EGF molecules on the λ surface. The ability of the *Dam15* mutation to be complemented by the *D::EGF* fusion was dependent on the tolerance of the translational allele of gpD produced by each *E. coli* suppressor strain within the phage capsid. Functionality of each translational allele was assessed by restored λ *Dam15* plating efficiency on each strain relative to the double suppressor (*supE supF*) host.

The translational fusion *D::EGF* was placed under an inducible strong promoter, λ *pL*, which is under the control of a temperature-sensitive λ CI857 repressor. Under this governance, *D::EGF* is repressed at 30 °C (very low expression level) and induced at 37°C. The plating efficiency in the Sup^-^ strain increased by a 10^3^-fold when *D::EGF* was expressed in trans, indicating that the fusion is able to compensate partially for the *Dam15* mutation and be incorporated into the capsid to form a viable phage (**Supplementary Table S2**). Isogenic suppressor strains of W3101 (*supD, supF*) substitute an amino acid in place of the amber stop signal generating a readthrough translational allele of gpD. SupD and SupF isogenic amber suppressor strains will substitute serine (gpDQ68S) and tyrosine (gpDQ68Y), respectively, in place of the stop codon at the 68^th^ codon. The gpDQ68S translational allele is very poorly tolerated in capsid formation as demonstrated by very poor plating efficiencies of λ *Dam15* on the SupD derivative (101). We similarly noted poor suppression of the *Dam15* mutation by SupD in this study, where the efficiency of plating of SupD was observed to be marginally lower than that of Sup^-^ control. Complementation of the *Dam15* mutation by *D::EGF* fusion however, was able to restore plating in SupD hosts by more than 10^3^-fold. In contrast, SupF is highly efficient at suppressing the *Dam15* mutation, and as such, no further increase in plating efficiency was noted by the expression of the *D::EGF* fusion.

### EGF-targeted λ phage infiltrate human colon carcinoma spheroids

Having established that the λ phage is capable of penetrating connective tissue stroma and ECM, and that EGF may be coupled to gpD capsid protein as a targeting ligand, we examined the interaction of EGF-targeted λ phage with HT-29 spheroids. HT-29 cells form tight spheroids that can resist the cytotoxicity of even small-molecular-weight compounds (97). They are therefore ideal to investigate the accumulation of λ phages in a 3-dimensional human tumor model in which the neoplastic epithelial cells form tight cell-cell contacts. Furthermore, the receptor for EGF is an appropriate target as it is overexpressed in HT-29 cells and has been implicated in this type of carcinoma. The two targeted λ phage preparations produced had two degrees of EGF display: EGF^Lo^ and EGF^Hi^ λ variants, which had decorations of approximately 90 and 147 EGF molecules/surface, respectively.

**Figure 4:**
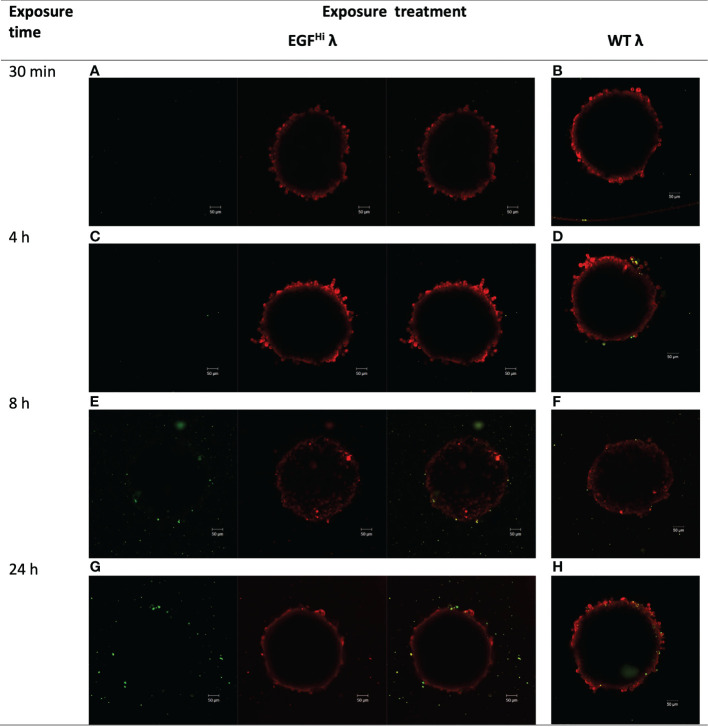
λ phages accumulate in HT-29 colorectal cancer spheroids, and the accumulation/distribution is altered by EGF displayed on the capsid surface. HT-29 spheroids (d6, ~300 μm diameter) were treated with WT λ or EGF^Hi^ λ phages, each tagged with Alexa Fluor488 (green). Spheroids were washed to discard unbound phages and counterstained with CellTracker™ Red prior to imaging by confocal microscopy. Panels show the images at the successive timepoints noted, for EGFHi λ (panels **A, C, E, G**) or WT λ (panels **B, D, F, H**). Images are from a z plane 64 μm below the surface of the spheroids, except for panel E (42 μm).

First, HT-29 colon carcinoma spheroids were treated with 10^8^ PFU of AF488-tagged EGF^Hi^ and WT (EGF^–^) λ phages for up to 24 h. Both AF488-EGF^Hi^ and AF488-WT λ adhered to the HT-29 cells and accumulated so as to be resistant to rigorous washing in a time-dependent manner (**Figure 4**), indicating that both native and EGF-decorated phages could infiltrate the outer cellular layers of these dense CRC spheroids as with the fibroblastic stroma model.

With AF488-WT λ there was punctate interaction with surface HT-29 cells by 4 h but no substantial increase thereafter and no focal accumulation (**Figure 4D**). The EGF targeting of the AF488-EGF^Hi^-λ was associated with a delay in the association of phages with the cancer spheroid, which only became significant and clearly observable after 8 h (**Figure 4E**). However, the interaction of AF488-EGF^Hi^-λ with spheroids continued and reached a higher level at 24 h (**Figure 4G**). As well, there were abundant small structures separate from the spheroid itself but which sedimented along with the spheroids in the washes (**Figure 4G**).The association of these particles with the vicinity of the spheroid itself suggested that they might be AF488-EGF^Hi^-λ bound to EGFR on small membrane vesicles shed from the HT-29 cells, either as part of their maturation process or as a consequence of the initial interaction of the AF488-EGF^Hi^-λ with intact, viable cells at the spheroid surface.

In view of the difficulty in evaluating spheroid uptake against this background of subcellular material, we adopted a quantitative approach to evaluate phage uptake into HT-29 cells in spheroids, and the impact of EGF targeting of the λ phage.

### EGF-targeted phage λ accumulate in human colon carcinoma spheroids

Following exposure of mature spheroids over the same timeframe as in **Figure 4**, we extensively washed the spheroids to remove weakly-bound phages and then used a 2-stage extraction process to recover the phage particles either (i) constrained within intercellular spaces or (ii) taken up or embedded inside cells. Intact, functional λ phages were enumerated for PFU, and we corrected data for the loss of phage infectivity due to treatment duration at 37 °C.

**Figure 5:**
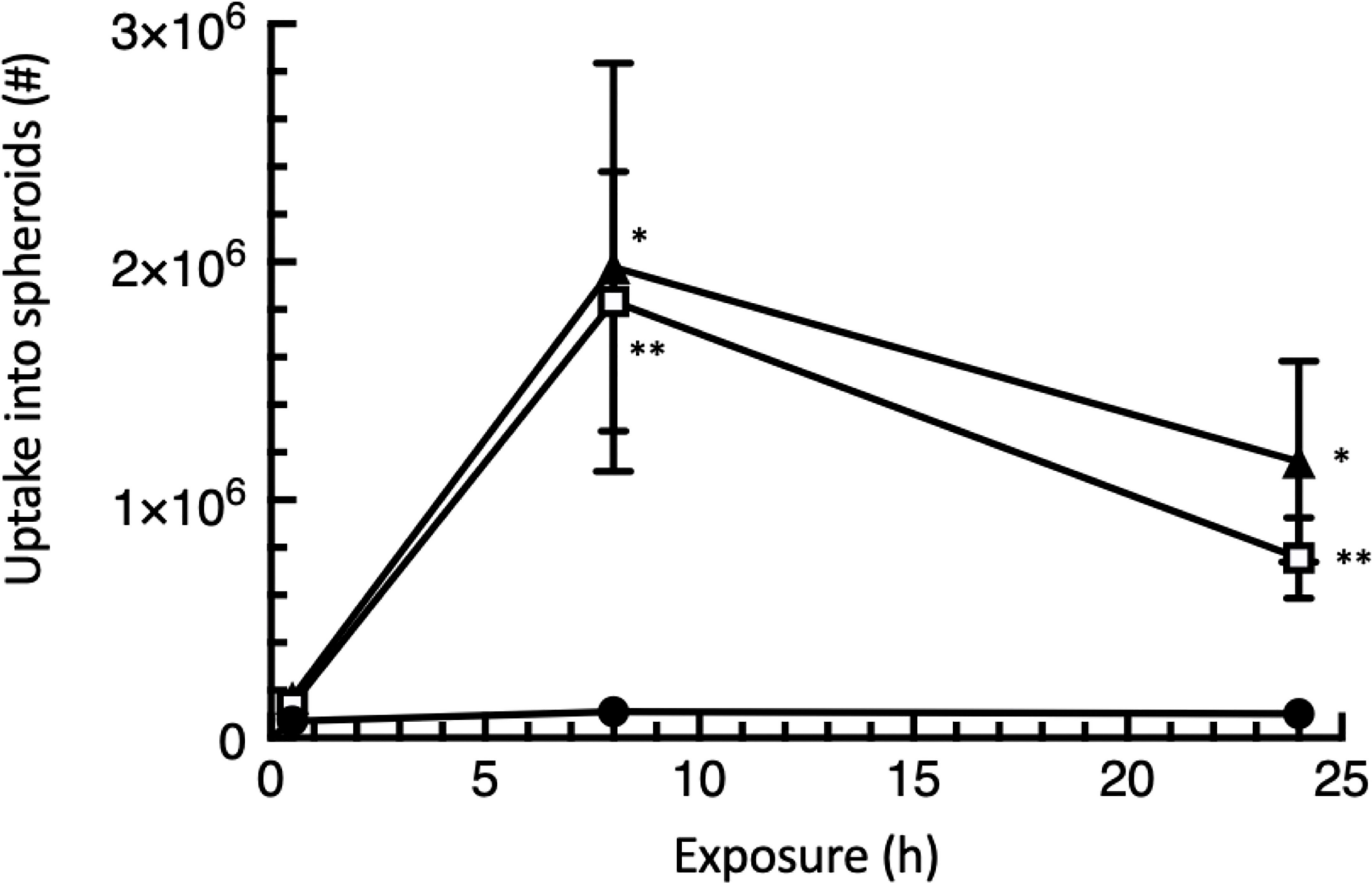
EGF decoration of λ phage substantially enhances penetration of into HT-29 colorectal cancer spheroids. HT-29 spheroids were exposed to 10^8^ phages for the times shown and the total that had infiltrated into the spheroids then recovered by fully dissociating the spheroids and subjecting the cells to complete lysis. Each of the data points has been adjusted to allow for the loss of inherent phage infectivity due to the treatment duration. The data mean values ± SEM, calculated from 3 independent experiments each with 3 replicates. An uptake of 2 x 10^6^ phage corresponds to 2% of input. The symbols represent l, WT λ; **□**, EGF^Lo^ λ; s, EGF^Hi^ λ. Significant differences from WT: * P<0.05; ** P<0.01.

We found that the association of WT λ within the looser surface cell layers of HT-29 spheroids was of low affinity and phages were removed by rigorous washing (**Figure 5**). The maximum persistent accumulation of phages was negligible, only 0.11% of input at the 8 h timepoint, the highest accumulation reached. We therefore conclude that the association of WT λ with the cancer spheroid, although rapid, is a weak association and therefore unlikely to offer great promise to infiltrative therapeutic strategies using native (untargeted) phages.

However, authentic persistent infiltration of both EGF^Hi^ and EGF^Lo^ *-λ* phage particles was readily observed (**Figure 5**), showing that the EGF targeting strategy was successful to enable persistent uptake. EGF-targeted phages were recovered at 16.8 to 18.1-fold (8 h) or 7.4 to 11.4-fold (24 h) compared to the uptake of WT λ. The uptake of EGF^Hi^-λ was only marginally greater than that of EGF^Lo^-λ in these experiments. This suggests that the lower decoration level (less than 100 EGF molecules on each surface/phage) which is readily attainable, is sufficient for the outcomes seen here.

Our quantitative measurements of penetration gave lower values for EGF-targeted phage at 24 h compared with 8 h, in contrast with observations in microscopy (**Figure 4**). However in the approach here, we were measuring functional λ phages using the PFU assay rather than the fluorescent tag, so we speculate that the finding reflects a loss of phage functional integrity with time, within the cell mass of the cancer spheroid.

### EGF-targeted phage λ is taken up intracellularly within HT-29 carcinoma cells

The two-phase extraction of extensively-washed spheroids allowed us to distinguish between phages recovered from tissue interstitial (*intercellular*) spaces and those present within the constituent cells (*intracellular*), accessible only by cellular disruption. Our approach quantified intact, functional phages as defined by their ability to form plaques in bacterial monolayers, corrected for the decline in intrinsic activity due to incubation at 37°C. **Table 1** shows the results normalized relative to WT, for both EGF^Hi^-λ and EGF^Lo^-λ.

**Table 1:** EGF targeting of λ phage enhances uptake into HT-29 colorectal cancer spheroids and facilitates cellular entry.

Time of exposure	Phage Treatment					
	WT λ		EGF^Lo^-λ		EGF^Hi^-λ	
	*Intercellular^1^*	*Intracellular^2^*	*Intercellular*	*Intracellular*	*Intercellular*	*Intracellular*
30 min	1.0^#^	1.0	1.60 ± 0.54	2.31 ± 0.96	2.31 ± 0.88	2.51 ± 1.31
8 h	1.0	1.0	19.68 ± 5.91**	19.54 ± 7.39*	19.88 ± 8.71*	23.14 ± 9.46*
24 h	1.0	1.0	5.23 ± 1.19**	53.59 ± 19.99*	5.55 ± 2.00*	40.23 ± 23.72

Mature spheroids were incubated with WT λ, EGF^Lo^-λ or EGF^Hi^-λ at the doses indicated. At the end of the time period of 30 min, 8 h or 24 h the spheroids were assessed for uptake into the interstitial fluidic spaces (‘intercellular’), or presence within extensively-washed cells dissociated from the spheroids (‘intracellular’). Phage counts were corrected for predicted reduced infectivity in PFU assays due to maintenance at 37 °C (**Supplementary Table 3**). Data are mean values ± SEM from 3 independent experiments after normalization to WT. As indicated, data have been normalized to WT as 1.0. Statistical analysis by 2-way ANOVA on the data before normalization showed significant differences (P<0.01 to P<0.05) based upon both phage type and exposure time, compared to relevant WT value.

^1^Phages recovered from the interstitial spaces of dissociated spheroids

^2^Phages recovered from extensively-washed and disrupted spheroid cells.

^#^These data have been normalized to WT.

*P<0.05; **P<0.01.

The data again show that in addition to much greater preferential uptake, there is a preferential shift to the intracellular compartment with EGF decoration. For EGF^Lo^-λ there is no shift in the distribution within the spheroid at 8 h, but at 24 h the distribution of liganded phage is biased more than 10-fold in favor of being in the intracellular compartment, showing active cellular uptake compared to the native phage. The same substantial difference is seen at 24 h for EGF^Hi^-λ, and given the 16% higher value it may be that with the greater EGF decoration the shift to the intracellular compartment is occurring earlier at ~8 h, although further work is needed to see if this reaches statistical significance. Nevertheless, these data provide convincing evidence for the cellular uptake of EGF-targeted λ phage after entry into HT-29 CRC spheroids, and that active entry begins soon after 8h of initial exposure, which is the main phase of phage entry into the spheroid.

### EGF-λ phages are not directly cytotoxic to HT-29 cells

**Figure 6:**
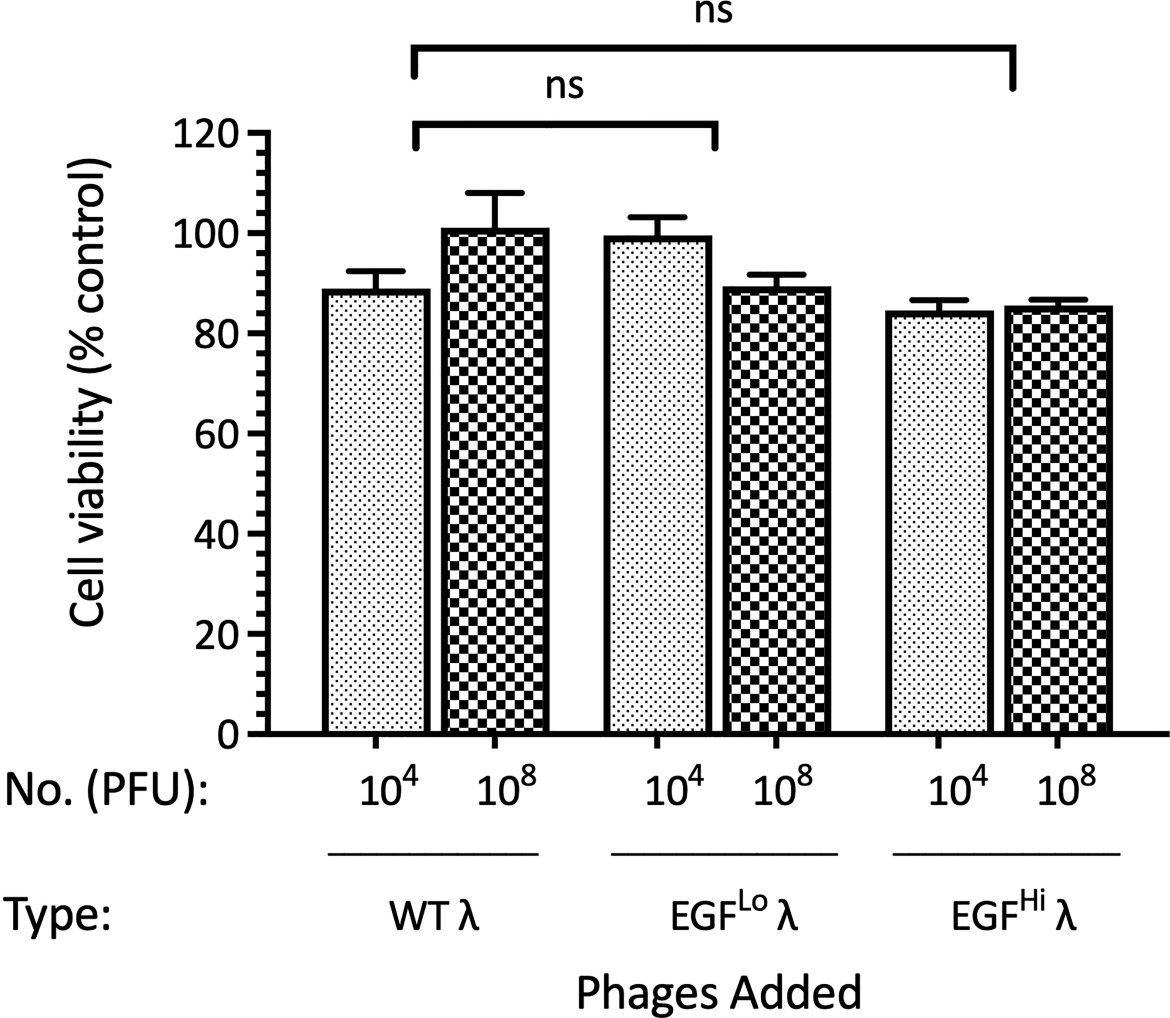
WT and EGF λ phages are not cytoxic for HT-29 cells grown in monolayer culture. HT-29 cells were established in monolayer for 72 h and then treated with 10^4^ or 10^8^ PFU of WT, EGF^Lo^ and EGF^Hi^ λ phages for a further 24 h. Cell viability was then assayed by the MTT assay and expressed relative to untreated cells. Data are expressed as mean ± SEM of 3 independent experiments, each with 3 culture replicates. Neither comparison with controls or comparison of EGF-displaying λ phages with WT (shown) showed a statistically significant difference. ns, not significant.

Given the ability of EGF-λ phages to both penetrate HT-29 cancer spheroids and enter cancer cells we wished to know whether the targeted carrier phage was directly cytotoxic to these cancer cells. We first evaluated both cytotoxicity and potential interference with cell metabolism using the MTT assay, which measures mitochondrial succinate dehydrogenase (SDH) activity (96). HT-29 cells growing in a monolayer were exposed to either low (10^4^ PFU) or high (10^8^ PFU) concentrations of both WT and EGF-targeted λ phages for 24 h and SDH activity then evaluated. As indicated in **Figure 6**, there was no loss of viability (or mitochondrial function) due to direct exposure of HT-29 cells with any of these preparations, and no significant differences between the results with the EGF-decorated and WT λ phages. We therefore conclude that these phages, without payload, are not directly cytotoxic to the HT-29 cells, regardless of whether they have the EGF ligand attached, or not.

### EGF-λ phages lead to a reduction in the ATP of HT-29 cells in spheroids

**Figure 7:**
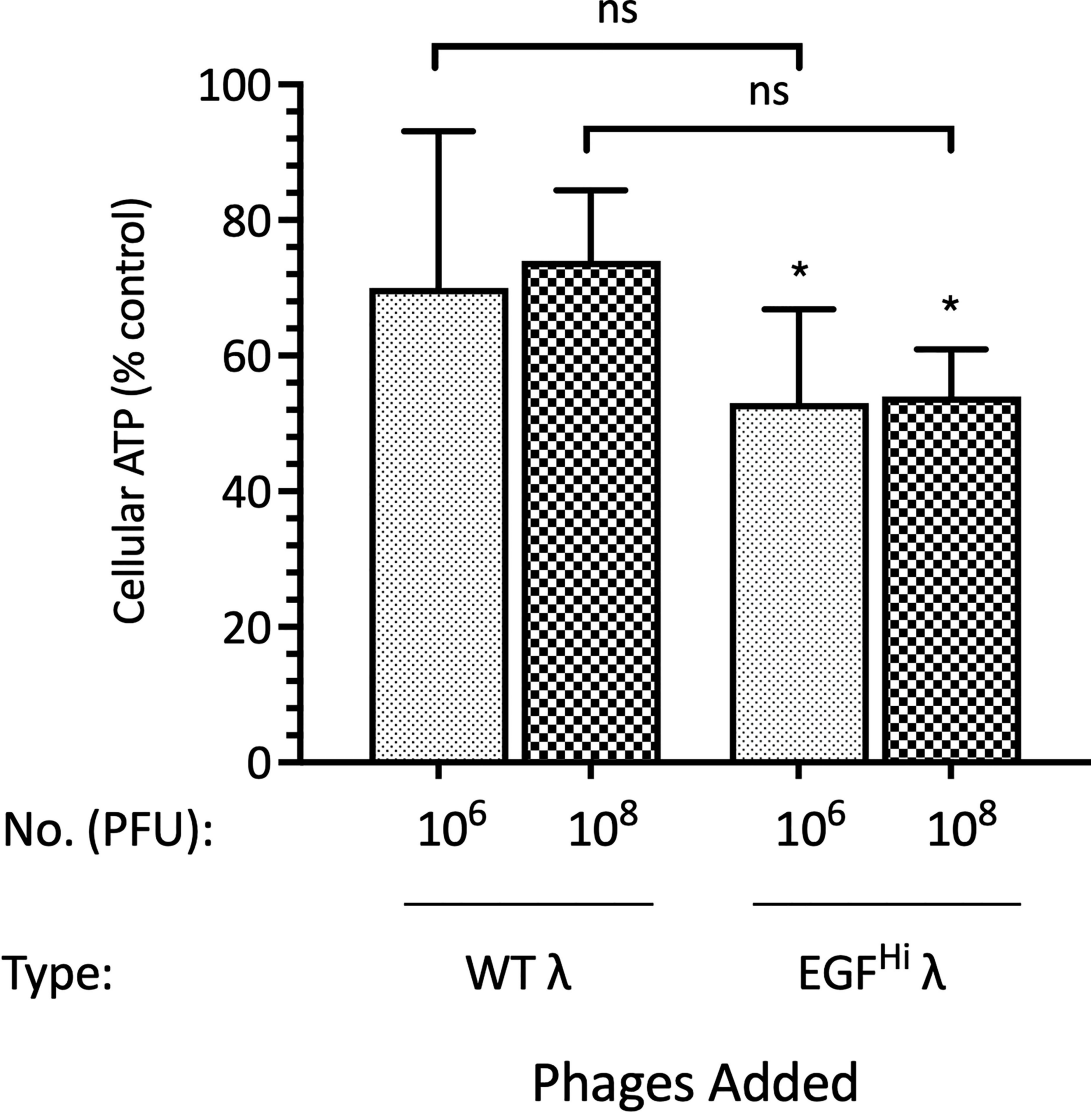
EGF decoration of λ phages leads to a reduction in the ATP of HT-29 cells in spheroids. HT-29 spheroids (d7) were treated with 10^6^ or 10^8^ PFU of WT λ or EGF^Hi^ λ phages for 24 h. The ATP content of spheroids was then determined. Data are expressed relative to spheroids in standard growth medium, as mean ± SEM of 3 independent experiments, each with 3 culture replicates. Significantly difference from control, * P<0.10; ns, not significant.

Despite there being no evidence for direct toxicity of the phage preparations against the cells in monolayer, we used a commercial assay of putative cytotoxicity to evaluate the apparent viability of the spheroid cell population after exposure to the phages. The assay measures total cellular ATP. While variability was high in these assays we noted a significant effect of EGF^Hi^ phages in depressing the cellular ATP levels in HT-29 spheroids, but no effect that reached significance for WT phages (**Figure 7**).

### EGF-targeted λ phages inhibit early stages of spheroid growth and formation

#### A. Treatment during initial aggregation of the cancer cells

**Figure 8:**
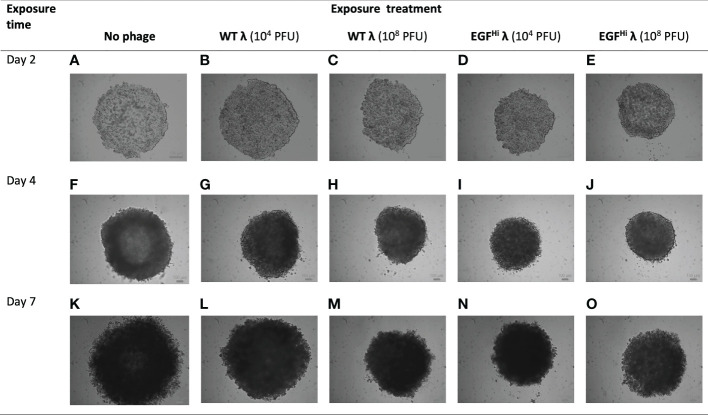
Addition of WT λ and EGF^Hi^ λ particles at initial cell aggregation reduce the size but do not compromise spheroid formation. WT λ **(B, C, G, H, L, M)** and EGF^Hi^ λ **(D, E, I, J, N, O)** phages at two doses (10^4^ or 10^8^ PFU) were added to the agarose surface at the same time as seeding with HT-29 cells. Initial cell aggregation and resultant spheroid formation were imaged up to d7 with a bright-field microscope, showing full-thickness spheroids and compared to controls **(A, F, K)**. Representative photographs from one series in 3 independent experiments. Individual scale bars are shown.

In our first approach we included phages at the time of seeding of HT-29 cells onto agarose. The initially-dispersed cells normally settle onto the non-adherent agarose and over the initial 2 d aggregate into a shallow disc, which subsequently folds in and merges from the periphery to form a fully intact spheroid around 7 d (97). We added WT-λ, EGF^Lo^-λ or EGF^Hi^-λ phages at either 10^4^ or 10^8^ PFU/culture at the time of seeding and observed the outcome (**Table 2**; **Figure 8**).

**Table 2:** EGF-targeted λ phage inhibit the growth of HT-29 colorectal cancer spheroids.

Addition	Number added (PFU)	Diameter (µm)	Difference from control	Difference from WT
Control	–	1,533 ± 47		
WT λ	10^4^	1,473 ± 202	n.s.	
	10^8^	1,410 ± 35	*	
EGF^Lo^-λ	10^4^	1,509 ± 52	n.s.	n.s.
	10^8^	1,198 ± 19	***	***
EGF^Hi^-λ	10^4^	1,329 ± 54	***	n.s.
	10^8^	422 ± 28	****	****

HT-29 spheroids were grown without addition (‘control’) or with addition of phages as described, at the time of addition of the cells onto agarose. Spheroid diameters were measured after growth to d7. Data are mean values ± SD (n=3). Statistical analysis by 2-way ANOVA showed differences based upon phage type and number added, with individual comparisons as noted.

* P<0.05; *** P<0.001; **** P<0.0001; n.s., not significant.

Relative to the control situation with no phage added, the highest addition of WT-λ and EGF^Lo^-λ significantly reduced spheroid diameter at d 7 (**Table 2**).The effect was more noticeable with EGF^Hi^-λ, with significant reductions in ultimate spheroid size being achieved with additions of 10^4^ or 10^8^ PFU of EGF^Hi^-λ. This reduction in size was also observable in microscopy (**Figure 8**). The intermediate stage of spheroid formation, in which the aggregate has a cross-sectional profile similar to that of an erythrocyte, can be seen in the d 4 no phage control (**Figure 8F**). Again, low concentrations (10^4^ PFU) of WT λ had no effect on spheroid formation but higher concentrations (10^8^ PFU) of WT λ and both concentrations of EGF^Hi^-λ phages interfered with initial aggregate formation and subsequent growth, leading to a smaller spheroid at d 7. The measured diameter ranges respectively were: d 4, 732-772 μm vs 949-1025 μm; d 7, 823-968 μm vs 1210-1309 μm). All of the spheroids retained their typical mature final phenotype, even though sizes varied, confirming that any deviation spheroid evolution process would not be due to λ phage effects on initial cell aggregation.

#### B. Treatment after initial aggregation of the cancer cells

**Figure 9:**
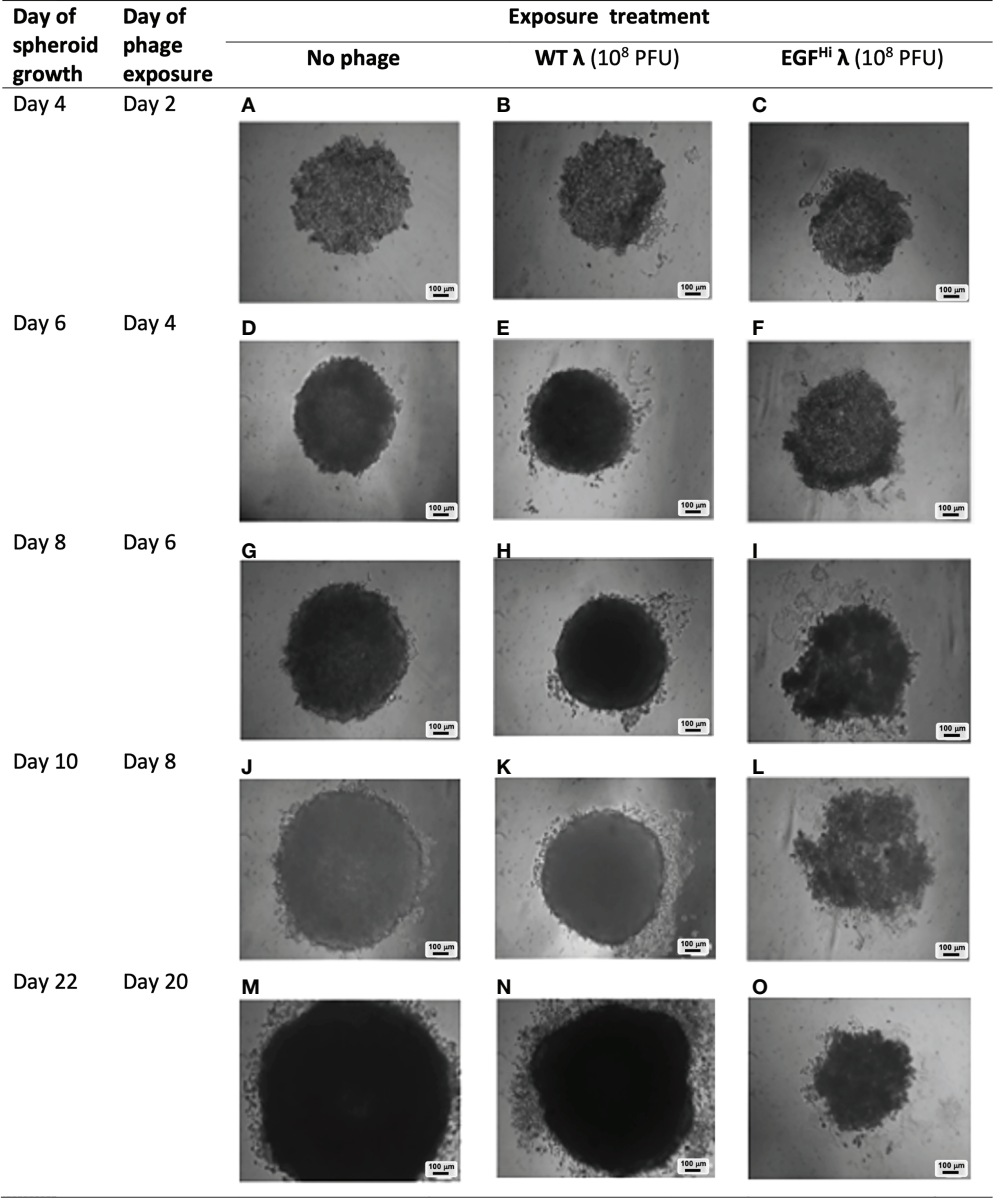
Persistence of effect of EGF-decorated phages on spheroid development. WT λ **(B, E, H, K, N)** or EGF^Hi^ λ **(C, F, I, L, O)** phages (10^8^ PFU) were added to partially-formed HT29 spheroids at 48 h after seeding and followed for a further 20d as shown, and compared to controls **(A, D, G, J, M)**. The precise morphologic findings were varied over 3 independent experiments but this shows a representative example of how the EGF λ phages had a persist disruptive effect on spheroid formation that was clearly evident out to d8 after phage addition and led to the ultimate formation of a significantly compromised spheroid. This was not evident with WT λ, or with λ phages with lower EGF decoration (EGF^Lo^ λ, not shown). Images are brightfield of spheroids *in situ* on agarose, and scale bars are shown.

We next added our λ preparations (WT and EGF^Hi^) at 10^8^ PFU to partially-formed (d 2) HT-29 spheroids, so that the phages encountered the cells post aggregation as the spheroids were developing further (**Figure 9**). We compared effects with the natural evolution of the spheroids (control condition) over a full period of 22 d, the longest that we routinely maintain these spheroids in original culture.

We observed no consistent change to spheroid formation over the first 4 d of phage exposure with either of our λ preparations (**Figure 9**). At d 6 there was change apparent in spheroids exposed to EGF^Hi^-λ, which began to exhibit an irregular morphology and be noticeably less dense than other spheroids. By d 8 the HT-29 spheroids treated with EGF^Hi^-λ showed clearly aberrant formation, with the spheroidal integrity lost, cell clumps dissociating and sparse regions in the cellular aggregates. By d 20, the disruption in spheroid formation had resulted in a spheroid that was markedly smaller in diameter than the control spheroid, and less dense, although had regained more of its usual spheroidal appearance. In contrast, neither the WT λ (**Figure 9**), EGF^Lo^-λ, nor lower doses (10^4^ PFU) of the phage preparations (data not shown) exerted any observable effect on the final spheroids.

#### C. Treatment of fully mature spheroids with phages

**Figure 10:**
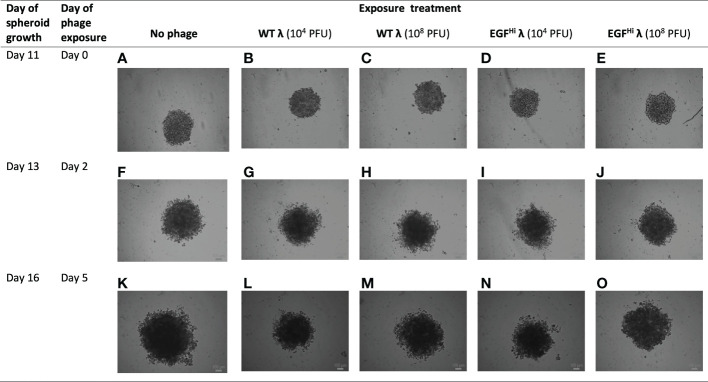
Addition of λ phages to fully-formed HT-29 spheroids does not impede tumor expansion. WT λ **(B, C, G, H, L, M)** and EGF^Hi^ λ **(D, E, I, J, N, O)** phages at two doses (10^4^ or 10^8^ PFU) were added to fully-formed but small dimension HT-29 spheroids at day 11 after seeding. Spheroid development was imaged for a further 5 d. with a bright-field microscope, showing the full-thickness spheroids and compared to controls **(A, F, K)**. Representative photographs from one series in 3 independent experiments. Scale bar: 100 μm.

Finally, we added our phage preparations to fully-mature spheroids grown to d 11 (**Figure 10**). The HT-29 spheroids continued to expand modestly over this period as expected and began to show early signs of disintegration as is typical for these spheroids (their sizes being such that the central portions of the spheroids are hypoxic). However, in no case (WT λ or EGF^Hi^-λ; 10^4^ PFU or 10^8^ PFU) was there a consistent change in the integrity or size of the spheroids above the general spheroid variability. Thus, neither native nor EGF-decorated λ phages appear to affect mature spheroids.

## Discussion

Given the substantial advantages of phage technology for the design of novel therapeutics (104) we investigate the use of engineered bacteriophages in cancer therapeutic applications. In particular, we see the potential for interference in some of the behaviors of infiltrating immune effector cells as well as neoplastic cells that favor metastasis and are influenced by the features of the 3-dimensional tumor environment, such as low-molecular-weight tissue mediators (22, 23, 25, 27, 96, 97). For this to be done we need first to determine whether phage constructs can find their way into the dense tissue of a tumor and interact with cancer cells to deliver therapeutic cargos.

We approached this question in stages using multicellular spheroids to generate representative 3-dimensional models of the stroma and tumor tissues (97, 105). We first used spheroids of NIH3T3 cells, which are a multipotent mesenchymal cell line with predominantly fibroblast character and serve well as a model for stromal components of the tumor capsule and septa that might impede phage penetration. We found that WT λ phages (covalently tagged with a fluorochrome) adhered to spheroids and became bound or entrapped within the outer cellular layers and matrix of the model stroma. Confocal images showed λ phage particles within periphery of the fibroblast spheroids at times beginning at 6 h, a time course of ingress that would be acceptable for therapeutic purposes. Over the relatively short timeframe of 24 h, there was evidence for entry of the tagged particles in a concentrated form in the outer 2-3 cellular layers, and potential dissemination to deeper levels that needs to be further studied.

Fibroblastic cells, which tightly adhere to each other in the spheroid as they do in stromal tissue, would therefore not constitute a barrier to access of λ phage. The ability of phage particles to penetrate through solid tumours in patients will depend on a variety of factors including size and morphology of the phage and biochemical properties of the phage capsid that may affect its interactions with the ECM (106, 107). Whether there are productive interactions between λ phages and the ECM formed by the fibroblasts is a matter of further investigation; but if so this would favor local accumulation of phages in a tumor.

We next studied phage infiltration into spheroids modeling the neoplastic parenchyma, which is a greater barrier due to tighter associations between epithelial cells than fibroblasts (97). We also enabled cancer cell-selective targeting by expressing EGF at the capsid surface to allow binding to the EGF receptor (EGFR). The EGFR is a transmembrane receptor tyrosine kinase in the ErbB family of receptors and is most highly expressed on epithelial cells. It has been implicated in the progression of wide range of epithelial tumours, for which increased EGFR activity enhances growth, invasion and migration (108, 109). Although EGF : EGFR interaction is essential for normal maintenance and renewal of epithelial cell populations, the overexpression and hyperactivation of EGFR contributes to tumor progression, rendering it an important biomarker and target for anticancer therapeutics (110). EGF has been used in directing other types of vectors for a targeted approach in gene therapy applications (111, 112). Human EGF has been shown to retain its ability to bind to EGFR when fused to fd filamentous phage (112) and the display of EGF on phage particles can direct the phage vectors to recognize EGFR-expressing cancer cells (113). EGF itself is well suited as a ligand for targeting due to its stability. The displayed growth factor is unlikely to have unfolded in the conditions used in this study as recombinant human EGF unfolds only under extreme pH conditions (pH < 4 and pH > 8) or temperatures higher than those encountered here (114). In this study we displayed EGF by translational fusion to λ phage gpD capsid proteins using the dual-phage display system we previously described (101).

We used HT-29 CRC cells to model colorectal cancer tissue. HT-29 cells overexpress EGFR and are representative of wildtype-*KRAS* and *BRAF-*mutated tumours, which have been reported in approximately 5-15% of patients with colorectal cancers (115, 116). We demonstrated the time-dependent trapping of **λ** phages (both WT and with EGF displayed) in the peripheral layers of HT-29 spheroids. Phages accumulated to a lesser degree within HT-29 spheroids than in NIH3T3 spheroids after 24 h of treatment, which could be expected due to the tight adherence junctions between the epithelial cells comprising the HT-29 spheroids (65). Surface decoration with EGF slowed the initial penetration into HT-29 spheroids over the first 8 h, most likely due to immediate capture of the EGF ligand by EGFR on the most outer surface. The greater size of the EGF-decorated phage, which for the EGF^Hi^-λ preparation reflects a substantially greater molecular mass (ΔM_r_ ~ 890K) is also likely relevant. However, decoration with the 6-kDa growth factor or a comparable small polypeptide ligand is preferable to decoration with a larger targeting ligand. We have shown that phages maximally decorated by a large protein such as eGFP (33-kDa) can be as large as three times greater in diameter than WT phages (101). However, this does not indicate a concern in terms of overall access; the accumulation of EGF-decorated phage was greater than WT and involves an active process.

Quantitative analysis with rigorous washing of the spheroids showed that stable penetration of WT λ phages was in fact limited, and EGF decoration greatly enhanced and stabilized the accumulation within the cancer spheroid. In these studies, there was very little difference between uptake of the EGF^Lo^ and EGF^Hi^ variants, suggesting that the lower decoration level of less than 100 EGF molecules/phage is sufficient for efficient entry into the spheroid for delivery of a toxic payload. Higher ligand densities may encounter limits due to receptor saturation, as we have found in other contexts (14, 117).

The numbers of EGF^Lo/Hi^-λ phages recovered in this approach declined from 8 h to 24 h, in contrast with the observations from confocal microscopy, for which there was an increase in the fluorescence intensity from tagged EGF^Hi^-λ over this period. The quantitative method measures intact, functional phage by plaque-forming assay, whereas microscopy simply depends on sufficient residual macromolecular material to retain the fluorescent tag *in situ*. Hence, the observations can be rationalized if the EGF-λ becomes taken up and at least partially degraded by cells inside the spheroid after ~8 h.

Separation of EGF-λ phages from intercellular and intracellular compartments confirmed a ~20-fold overall increased uptake compared with WT λ. Moreover, by 24 h there was enrichment in the *intracellular* fraction by a factor of 10.2 for EGF^Lo^-λ and 7.2 for EGF^Hi^-λ. This clearly shows cellular uptake between 8 - 24 h and is consistent with the suggestion that a substantial fraction of the uploaded dose has undergone at least partial degradation or other inactivation that is seen in measured plaque-forming ability.

Our results are consistent with observations in other systems. The finding that EGF-λ were internalized more readily than untargeted phage agrees with the results of Kassner and colleagues who observed a similar distinction between EGF-displaying M13 phage and WT phage in transfection efficiency of COS-1 cells (113). Menon and colleagues showed that 15-60 min may be sufficient for cellular uptake of PEP-2 phages displaying ligands into ECV304 cells (118).

We wished to know whether λ phage, either WT or with EGF as a targeting ligand, had inherent cytotoxic activity or instead are likely to be a neutral vehicle for delivery of focused genetic payloads. We found that λ phages, with or without EGF decoration, were not cytotoxic for HT-29 cells in monolayers, nor had any effect on overall mitochondrial function, as assessed using an acute viability assay with succinate dehydrogenase as a readout. This is consistent with the finding that other phages have no cytotoxic effect on these cells (119). We did find evidence for an effect using an assay that is presumed to reflect viability in spheroids (see **Figure 7**). However, interpreting this as a loss of viability of near 50% over 24 h is both biochemically implausible given the ligand and inconsistent with the MTT assay (**Figure 6**) and subsequent timed morphological analyses (**Figures 8**–**10** and **Table 2**). We think it more likely that this reflects changes in energy utilization in response to phage exposure, which we have shown involves uptake and likely degradation of phages. The assay itself measures ATP levels and as these mature spheroids have substantial hypoxia and low glucose levels, replenishment of ATP may be a limiting factor.

Changes in the ATP assay raised the possibility that EGF-λ might interfere with development of neoplastic tissue, as modeled with our spheroids, independently of any direct cytotoxicity. This might interact beneficially with any anticancer therapeutics, either those encoded in the phage itself or provided independently in combination treatment. For example, partial dissociation of the cell population might facilitate drug access or reduce the resistance associated with hypoxia or cell adhesion mechanisms. We therefore examined the effect of additions at different times on spheroid evolution, which is itself a mimic of the growth of microdeposits.

Addition of WT or EGF^Hi^-λ phage to fully mature spheroids had no effect on spheroid expansion or appearance, suggesting it would be unlikely for this approach to have a direct beneficial effect on established tumours, without a toxic payload. However, the addition of EGF^Hi^-λ at the time of cell seeding (comparable to initiation of secondary deposits from initial disseminated cells) interfered with full development of cancer spheroids. Furthermore, addition of EGF^Hi^-λ 48 h after cell seeding on agarose, after cell aggregation and when the early cell clusters are proliferating and organizing into larger clusters, had a variable but at times dramatic effect on the fate of the spheroid. A slowly-developing effect on spheroid integrity led to significant dissociation of the cell mass over about a week, and the impact of this event was retained over a 3-week time course, leading to a much reduced and poorly-formed cell mass. This raises the possibility of a beneficial additive or synergistic effect of this delivery system as part of an antineoplastic strategy, interfering with the early seeding and expansion of microdeposits in regional establishments of secondary deposits. Collectively, our data also exclude concerns that the approach might in some way have a proneoplastic action that would make it difficult to use in an anti-cancer context.

Our findings suggest in principle that EGF-displayed/targeted λ phages may be valuable in delivery of toxic therapeutics of interventions that may release activity of local anti-tumor responses or immune checkpoint inhibitors. Our EGF-λ phages may themselves be able to disrupt early stages of CRC tumor metastasis formation. The latter effect might be due to the particular nature of the EGF signal delivered through the EGFR by the phage-EGF construct or may be a property of the λ phage itself when held in place at the cancer cell surface. Binding of EGFR by EGF-targeted phage may trigger inhibitory events similar to those for cetuximab and other mAbs that bind to the extracellular domain of EGFR (120–122). It is further possible that the effect of EGF-λ may depend on first taking up the decorated complex into the cancer cell, which will be a requirement for future delivery of a gene therapeutic. Phages can be taken up into mammalian cells *via* caveosomes and/or clathrin-coated vesicles, which are able to accommodate particles of at least 500 and 200 nm, respectively (123). Our data confirm that there is cellular uptake, based upon the need for cell lysis to recover a significant proportion of phages after incubation, and the loss of functionality most likely due to enzyme degradation. Alternatively, the phage itself, with or without partial activation of the EGFR, may interfere with aspects of the extracellular milieu such as ECM elaboration or structure, that can impact on tumor development.

In summary, we have shown that: (i) λ phages are able to pass through fibroblast and ECM layers that represent the stromal components of a tumor, (ii) enter and accumulate in dense tissue representing the CRC neoplastic parenchyma, (iii) EGF ligand, covalently bound to gpD capsid protein, favors entry within the cancer cell population, (iv) EGF-λ enters CRC cells in a model tumor at more than 50-fold the relative abundance of WT phage, (v) EGF-λ has the potential to interfere with the early formation and growth of cancer populations without the need for a toxic payload, and (vi) the effect of a single early exposure to EGF-λ persisted for the entire tissue progression (to d20) in our model CRC tumor system. We have therefore shown in principle the utility of targeted phage technology for infiltration of colorectal cancer tumor tissue (and by extension other solid carcinomas). The intermolecular mechanisms remain to be fully elucidated, but the approach should be translatable to patients *in vivo* and can be extended by insertion of a gene therapeutic to be locally expressed following cellular uptake of the phages. Such an intervention may enable new direct cytotoxic effects and/or dampen down immune escape mechanisms to allow greater host anti-tumor activity or facilitate the success of cell-based immunotherapies.

## Data availability statement

The original contributions presented in the study are included in the article/**Supplementary Material**. Further inquiries can be directed to the corresponding authors.

## Author contributions

RS and JB contributed to conception and design of the study. HH carried out experiments with assistance of D-WC and input from the other authors. Analysis of confocal images was by MF. The first draft of the manuscript was prepared by HH and extensively revised by JB and MF with contributions from the other authors. Statistical analysis was by HH and JB. All authors contributed to manuscript revision, read, and approved the submitted version.
