# Supplementary material for: EGFR-targeted bacteriophage lambda penetrates model stromal and colorectal carcinoma tissues, is taken up into carcinoma cells, and interferes with 3-dimensional tumor formation
Source: Front Immunol. 2022 Dec 16;13:957233. doi: 10.3389/fimmu.2022.957233 (PMC9800840; doi:10.3389/fimmu.2022.957233)
Supplement: Supplementary file 1 [file DataSheet_1.pdf]

SUPPLEMENTARY MATERIAL

Table S1: Summary of phages, cells and plasmids

Cell	Genotype	Source/Reference	Additional Information
BB4	*supF58 supE44 hdR514 galK2 galT22 trpR55 metB1 tonA* DE*(lac) U169*	Agilent Technologies, Inc.	This strain encodes a double suppression action (SupE and SupF) and serves as the positive control for the efficiency of plating (EOP) of λF7 samples.
W3101	*F-, galT22, λ-, IN(rrnD-rrnE)1, rph-1,*	CGSC #4467 [1]	This strain does not exert any amber suppression action and serves as the negative control for λF7 samples as λF7 is not viable on this host.
W3101 SupD	*F-, galT22, λ-, IN(rrnD-rrnE)1, rph-1, uvrC279::Tn10, serU132(AS),*	[2]	Amber isogenic suppressor SupD strain of W3101. W3101 SupD (*serU132*)
W3101 SupF	*F-, galT22, λ-, IN(rrnD-rrnE)1, rph-1, oppC506::Tn10 , tyrT5888(AS)*	[2]	Amber isogenic suppressor SupF strains of W3101. W3101 SupF (*tyrT5888*)
**Phage Strains**			
λF7	*λDam15imm21 cIts*	[3]	λ phage harbouring an amber mutation in the gene encoding gpD, and is able to form viable particles in the presence of the amber (UAG) stop codon at the position 68 of the 110 amino acid protein.

**Plasmids**			
pPL451 gpD::eGFP (pD::eGFP)	*pM-cI857-pL-cI857-pL-D::EGF-tL*	[2; 4]	Multicopy plasmid pPL451 that is under the control of a temperature-sensitive allele of the λ CI857 repressor. The eGFP sequence is fused to the C-terminal of the gpD capsid gene that is separated by an in-frame linked sequence p(TSGSGSGSGSGT) and a *KpnI* cut site.
pHH1		This study	Multicopy plasmid pPL451 that is under the control of a temperature-sensitive allele of the λ CI857 repressor. Similarly to pD::eGFP, the EGF sequence is fused to the C-terminal of the gpD capsid gene that is separated by an in-frame linked sequence p(TSGSGSGSGSGT) and a *KpnI* cut site.

[1] B.J. Bachmann, Pedigrees of some mutant strains of Escherichia coli K-12. Bacteriol Rev 36 (1972) 525-57.

[2] J. Nicastro, K. Sheldon, F.A. El-Zarkout, S. Sokolenko, M.G. Aucoin, and R. Slavcev, Construction and analysis of a genetically tuneable lytic phage display system. Appl Microbiol Biotechnol 97 (2013) 7791-804.

[3] Y.G. Mikawa, I.N. Maruyama, and S. Brenner, Surface display of proteins on bacteriophage lambda heads. J Mol Biol 262 (1996) 21-30.

[4] S. Sokolenko, J. Nicastro, R. Slavcev, and M.G. Aucoin, Graphical analysis of flow cytometer data for characterizing controlled fluorescent protein display on lambda phage. Cytometry A 81 (2012) 1031-9.

Table S2: The *Dam15* mutation can be complemented by *D::EGF* fusions in trans.

**Strain [+/-Plasmid][1]**	**Relative efficiency of plating (EOP)[2]**	**Increase in plating (compared to Sup-)**
Sup–	2.8×10^{-5}	**1.0**
Sup– [pD::EGF]	0.05	1.79×10^{3}
SupD	1.6×10^{-5}	0.57
SupD [pD::EGF]	0.12	4.29×10^{3}
SupF	0.23	8.21×10^{3}
SupF [pD::EGF]	0.10	3.57×10^{3}

[1]The *E. coli* suppressors are W3101 derivatives, and the plasmid pPL451 is under the temperature-regulated repressor CI857.

[2]All EOPs expressed as a mean of three assays with 3 replicates, determined using BB4 (SupE, SupF double suppressor *E. coli*) as the 100% plating control, and plated at 37°C.

Table S3: Prolonged exposure of phages at 37 ºC decreases λ phage infectivity.

	**EOP of λF7 incubated at 4ºC**	**EOP of λF7 incubated at 37ºC**	**EOP of D::EGF phages incubated at 4ºC**	**EOP of D::EGF phages incubated at 37ºC**
0 h	1.00 ± 0.02	1.00 ± 0.05	1.00 ± 0.05	1.00 ± 0.02
4 h	0.98 ± 0.01	0.91 ± 0.01*	0.95 ± 0.04	0.57 ± 0.02****
8 h	0.86 ± 0.05	0.73 ± 0.02***	0.82 ± 0.05	0.43 ± 0.04****
24 h	1.12 ± 0.02	0.46 ± 0.02****	0.86 ± 0.03	0.19 ± 0.08****
48 h	0.94 ± 0.03	0.35 ± 0.03****	0.86 ± 0.02	0.28 ± 0.04****

EOPs have been expressed as a mean ± standard deviation **of three independent assays, n=3, determined using BB4 (SupE, SupF double suppressor) as the 100% plating control and plated at 37 ºC. Comparisons of each phage types at two temperatures were made for each treatment period using the 2-way ANOVA test. Viability decreases of both phage types at 37 ºC were statistically significant for all treatment periods.**
